# Translation and Linguistic Validation of the Patient's Knee Implant Performance (PKIP) into Japanese

**DOI:** 10.1155/2024/6645361

**Published:** 2024-04-30

**Authors:** Fumiya Ohmasa, Yukihide Minoda, Daisuke Akahane, Kimberly A. Dwyer, Jim Lesko, Hideharu Shimizu

**Affiliations:** ^1^Johnson and Johnson K.K., 5-2, Nishi-Kanda 3-Chome, Chiyoda-Ku, Tokyo 101-0065, Japan; ^2^Department of Orthopaedic Surgery, Osaka City University Graduate School of Medicine, 1, 4-3 Asahi-Cho, Abeno-Ku, Osaka City 545-8585, Japan; ^3^DePuy Synthes Joint Reconstruction Inc., 700 Orthopaedic Drive, Warsaw, Indiana, USA

## Abstract

**Objective:**

The patient's knee implant performance (PKIP) is a patient-reported outcome measure, developed in the USA in English that evaluates knee functional performance before and after primary total knee arthroplasty (TKA). The PKIP assesses the level of satisfaction, confidence, and stability, while performing various activities, as well as the need for changing ways of doing activities. It comprises 24 items. The objective of this study was to present the methodology of the linguistic validation of the PKIP.

**Methods:**

The Japanese version of the PKIP was developed using a standard linguistic validation (LV) process. The LV involved the following steps: (1) conceptual analysis of the original version; (2) translation into Japanese using a dual forward/backward translation process; (3) review by an orthopaedics surgeon; (4) test on five respondents; and (5) proofreading.

**Results:**

The translation itself did not reveal major translatability issues, either cultural, semantic, or syntactic. Most of the activities listed (e.g., going up stairs, getting in/out of a car, and walking up a hill/ramp/incline) were easily translated. Only one activity was culturally sensitive and raised some discussion, i.e., “sitting down on a toilet,” since the style of Japanese toilets is different from the western style. Overall, the respondents well understood the questionnaire. However, the expression “how your knee is working with your body” used in the opening sentence was an issue for both the clinician and the respondents. A compromise was found by using a Japanese equivalent of “how your knee functions with your legs.”

**Conclusion:**

The rigorous translation process, which involved the collaboration of a minimum of thirteen people (sponsor, four translators, two coordinators (one in Japan and one in Europe), one clinician, and five respondents) enabled the production of a Japanese version of the PKIP conceptually equivalent to the USA English original.

## 1. Introduction

Total knee replacement or total knee arthroplasty (TKA) is a surgical procedure designed to relieve pain and disability in patients suffering mainly from severe osteoarthritis [1]. Katano et al. [2] reported based on the National Database of Health Insurance Claims and Specific Health Checkups that the total number of arthroplasties for all joints in Japan was 128,065 in 2014 and this number increased gradually to 146,189 in 2017, of which 56.3% was accounted for knee arthroplasty. Maradit Kremers et al. [3] estimated that the 2010 prevalence of total knee replacement in the total USA population was 1.52%, with an increase up to 4.55% among adults fifty years of age or older. They also showed that the prevalence was higher among women than among men and increased with age. Kurtz et al. [4] published the results of an interesting international survey (18 countries) exploring the use of primary TKA and subsequent revisions. Findings indicated that the demand for TKA has risen substantially over the past decade in countries around the world. Shichman et al. [5] projected an estimated TKA increase of 139% by 2040 and 469% by 2060 in the USA. Increase in TKA was shown in both males and females as well as across age groups. [6–8] Patients undergoing TKA are also more obese with 2029 projections being that 46% of the general population will be obese compared to 69% of the primary TKA population [9]. These incidence figures and changing demographics suggest that primary TKA and reason for revisions of TKAs will become a major healthcare issue in the future with need for developing appropriate outcomes tools to evaluate postsurgical outcomes for the changing patient population.

Lewis et al. [10] suggested that quantitative assessments of postsurgical knee motion provide sensitive measurements, but results are technical and may not be meaningful to patients. They also stated that although several knee-specific instruments exist (e.g., WOMAC [11], OKS [12], and KOOS [13]), no patient-reported outcome measure correlates function with improved stability, motion, satisfaction, and confidence. To address this gap, they undertook the development of the patient's knee implant performance (PKIP) questionnaire [10]. They created the PKIP around the concept of a “natural knee” or “natural” motion or movement after TKA, defined as stability, motion, ability with motion, satisfaction, and confidence with how an individual's replacement knee facilitates functioning. The PKIP was also built as a measure that could be used with existing knee-specific instruments to provide a more robust assessment of the patient's experience after knee replacement. It was developed in USA English, with a recall period as “the last week,” and consists of two versions, presurgical and postsurgical. Items used in these versions are identical. The main difference lies in the opening sentence, i.e., “the questions below ask about ways in which your knee is working with your body” (presurgery) vs. “the questions below ask about ways in which your knee replacement is working with your body to improve your performance” (postsurgery).

The PKIP comprises the following 24 items:

(i) Eighteen items assess confidence (*n* = 7), stability (*n* = 6), and changes in ways of doing activities (*n* = 5) and are scored on an 11-point numerical rating scale.
(ii) Four items are scored on a 5-point Likert-type scale:
  - Three on a frequency scale (never, rarely, sometimes, often, and always): they assess global awareness, confidence, and stability;
  - One assesses performance on an intensity scale (not at all, slightly, moderately, very, and completely).
(iii) Two items are using a 6-point Likert-type scale to assess satisfaction.

Coles et al. [14] undertook the psychometric evaluation of the PKIP in a population of USA patients with osteoarthritis and showed that the reliability, validity, and responsiveness of the PKIP support its use among patients undergoing primary TKA.

The objective of our study was to present the methodology of the linguistic validation of the PKIP.

## 2. Materials and Methods

### 2.1. Linguistic Validation Process

**Figure 1:**
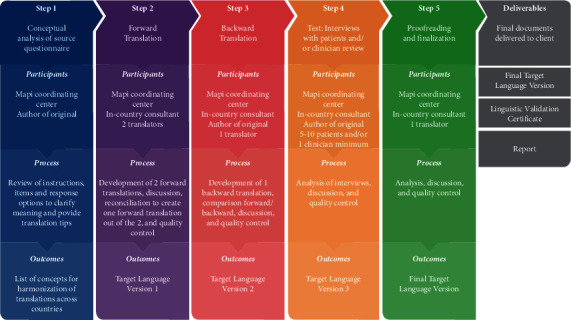
Standard linguistic validation process.

The translation process (i.e., linguistic validation [15]) used to develop the Japanese version of the PKIP followed the recommendations of the International Society for Pharmacoeconomics and Outcomes Research [16]. The process, conducted by a coordinating center (i.e., Mapi Language Services), consisted of five steps (Figure 1). The first step involved the conceptual analysis of the original USA English PKIP, in collaboration with the sponsor of the project, in order to provide the translation team with a document explaining the meaning of each instruction, item, and response options and suggesting terms to denote each concept. This was the basis for ensuring that the Japanese version of the PKIP was equivalent to the meaning of the original. Following that, the original USA PKIP was translated through a dual process of forward-backward translation and reviews by a local team leader and the project manager of the coordinating center who supervised the whole process. Two different Japanese versions were developed independently by two native Japanese translators living in Japan. These two versions were used to create one single translation called the reconciled version (i.e., step 2, forward translation step). This reconciled version was back translated into English (Step 3) and compared to the original USA PKIP to check for discrepancies and control the quality of the Japanese reconciled version. This backward translation step helped to refine the Japanese reconciled version. In step 4 (i.e., testing), the resulting Japanese version was reviewed by an orthopaedic surgeon (YM) and survey respondents who had a knee implant due to osteoarthritis check for the accuracy, appropriateness, understandability, and clarity of the wording. The process ended with a proofreading as a final quality check (step 5).

### 2.2. Survey Respondents

Subject selection criteria for a sample of five respondents were as follows: patients who had undergone a TKA due to osteoarthritis, native speaker of Japanese living in Japan, with balanced representation of gender (i.e., 2 males/3 females or 3 males/2 females), and of mixed education (i.e., a maximum of two participants with more than 15 years of education, i.e., a university degree). The respondents were recruited through an agency specialized in patients' recruitment.

### 2.3. Analysis

The linguistic validation history was reviewed to identify difficulties and problematic issues, as well as the solutions proposed to overcome them. The types of difficulties were categorized as cultural (C), idiomatic (I), semantic (S), or syntactic (Sy) (Table 1) [17, 18].

## 3. Results

### 3.1. Survey Respondents

Respondents' demographics are shown in Table 2. The respondents' average age was 61.2 years.

### 3.2. Analysis/Translation Issues

The translation itself did not reveal major translatability issues, either cultural, semantic, or syntactic. The questionnaire was well understood by the respondents. Most of the activities listed (e.g., going up/down stairs, kneeling on your knees, getting in/out of a car, walking on an uneven surface (such as a bumpy/broken sidewalk and sloping surface), getting up from a toilet, walking up a hill/ramp/incline, walking on slippery surfaces (such as wet grass and rainy streets), bending down to the floor (to pick up an object, reach an item in a low cabinet, etc.), and putting on your shoes) were relevant and easily translated. Only one activity was culturally sensitive and raised some discussion, i.e., “sitting down on a toilet,” since the style of Japanese toilets is different from the western style. After much debate within the team, it was decided to respect the original English and translate it.

On the semantic level, the expression “how your knee is working with your body” used in the opening sentence and translated literally was an issue for both the clinician and the respondents. The clinician had originally suggested to delete “is working with your body” and to replace it by “functions,” i.e., “how your knee functions.” However, the preclinician version was kept and tested on patients who all found that the expression “working with your body” was awkward and felt uncomfortable with it. After a thorough debriefing, the interviewer found out that the respondents' primary interpretation of “body” did not include legs and feet (to them, “body” would probably refer to the torso only). This was the main reason why they found that the wording was “too indirect” and difficult to understand. A solution was found to refer to the way the knee (or the knee replacement in the postsurgical form of the questionnaire) fits with the foot and to retain the concept of the synergy resulting from it (i.e., the result of the combination of the knee and foot on the performance of activities).

The clinician input was very helpful in developing expressions which sounded more natural in Japanese; for instance, for the translation of “peforming your day-to-day activities” or “how often you modify or change the way you do activities such as….”

The original English version and the Japanese translation version of the PKIP (pre- and postsurgical versions) are available as supplementary materials (available here).

## 4. Discussion

The adaptation of the PKIP questionnaire from USA English to Japanese did not reveal major semantic or cultural issues. It was important to utilize everyday language, yet remaining true to the original meaning of the items. This involved focusing on semantics and required extensive discussion about the meaning of each concept and the choice of the adequate words to convey it. This was accomplished through steady and continual discussion between the local team of translators, the local team leader, the central project manager, and the clinician involved in the linguistic validation process. The respondents provided input essential to adaptation of the PKIP so that the Japanese target population could easily understand each component. The PKIP was well accepted by the participants of the study, which supports the assumption that concepts assessed and identified during the development of the original PKIP [10] were equally relevant to the Japanese patients. The availability of the PKIP Japanese version will enable Japanese researchers to better explore the functional abilities, satisfaction, and confidence of their patients before and after surgery. It might also be an important measure to assess the need for or reasons to undergo TKA revisions [19] and compare to data reported from English-speaking countries [20, 21].

Published research on cross-cultural comparisons of self-reported performance of patients who undertook a TKA is still scarce [22–27]. The availability of the PKIP questionnaire in USA English and Japanese will encourage cross-cultural research in TKA and will be the first step to wider development and use in various cultural settings. International studies assessing differences of impact across cultures would be of great interest. They would enable cross-cultural comparisons and improve awareness, tracking, and management of impact on patients undergoing TKA in different cultures, thus providing opportunity for increased support.

## 5. Conclusion

The rigorous translation process, which involved the collaboration of a minimum of thirteen people (sponsor, four translators, two coordinators (one in Japan one one in Europe), one clinician, and five respondents) enabled the production of a Japanese version of the PKIP conceptually equivalent to the USA English original.

## Figures and Tables

**Table 1:** Categorization of translation difficulties.

Category	Definition
Cultural (C)	A word or formulation in the original is culturally loaded in the target context due to societal or religious customs (e.g., eating habits in Asian countries). The usage of certain words or phrases based on the culture of a given society may be improper in the target language. For instance, “starchy foods (e.g., potato and bread)” becomes “starchy foods (e.g., rice, pasta, and chapatti).”

Semantic (S)	Semantics concerns meanings, which are both denotative, i.e., the literal word (lexis), and connotative, namely, the set of cultural and/or subjective associations implied by a word in addition to its literal explicit meaning. This category includes lexical differences. For instance, English has a slightly larger lexicon than French. Therefore, some French words have no direct equivalent in English and would need the use of paraphrases. For instance, meet your responsibilities becomes meet your duties or meet your obligations.

Idiomatic/pragmatics (I)	The practicalities of how a language is used in its everyday context may be different between the source and target language. For example, one language may have more social registers than another (there are a number of different forms of addressing a person in Japanese, whereas English may only have one) and the idiosyncrasies of one language (repetitions, focus on particular words, use of particular idiomatic expressions, etc.) may not be found in another For instance, “I feel downhearted and blue” can be translated by an equivalent of “I feel downhearted and sad” or “I feel downhearted and depressed.”

Syntactic/grammar (Sy)	Syntactic difficulties correspond to specific aspects related to sentence structure, grammar, and punctuation. The structure and grammar of the source and target languages may diverge and may impact the identification of conceptually equivalent alternatives in a target language. For instance, the use of a verbal passive form in the original may not possible in some target languages, where active form is more current. For instance, the placement of the recall period might differ in some target languages. In English, it often goes at the beginning or end of the item, but in other languages, it might be grammatically necessary to place it in the middle of the item.

**Table 2:** Respondents' characteristics.

Respondent #	R^*∗*^1	R2	R3	R4	R5
Age in years	51	72	67	66	50
Sex (male/female)	Female	Male	Female	Male	Female
Occupation	Housewife	Office worker	Housewife	Office worker	Housewife
Study level	High school graduate	University	High school graduate	University	High school graduate

^*∗*^R: respondent.

## Data Availability

The data used to support the findings of this study are included within the article.

## Supplementary Materials

Supplementary Materials Supplement 1: the original English version of the PKIP questionnaire for presurgical. Supplement 2: the original English version of the PKIP questionnaire for postsurgical. Supplement 3: the Japanese translation version of the PKIP questionnaire for presurgical. Supplement 4: the Japanese translation version of the PKIP questionnaire for postsurgical.
