# Supplementary material for: Translation and Linguistic Validation of the Patient's Knee Implant Performance (PKIP) into Japanese
Source: Adv Orthop. 2024 Apr 30;2024:6645361. doi: 10.1155/2024/6645361 (PMC11074845; doi:10.1155/2024/6645361)
Supplement: Supplementary Materials — Supplement 1: the original English version of the PKIP questionnaire for presurgical. Supplement 2: the original English version of the PKIP questionnaire for postsurgical. Supplement 3: the Japanese translation version of the PKIP questionnaire for presurgical. Supplement 4: the Japanese translation version of the PKIP questionnaire for postsurgical. [file 6645361.f1.zip › Supplement 1. English PKIP (pre-surgery) (2).pdf]

PATIENT'S KNEE IMPLANT PERFORMANCE (PKIP) — Pre Surgical

The questions below ask about ways in which your knee is working with your body.
Please select only one answer for each question.

1. How often are you aware of your knee?

Never	Rarely	Sometimes	Often	Always
☐	☐	☐	☐	☐

The following questions should be answered thinking of your knee during the **last week**.

2. Overall, how satisfied are you with how your knee feels when doing <u>everyday activities?</u>

Very Dissatisfied	Dissatisfied	A little Dissatisfied	A little Satisfied	Satisfied	Very Satisfied
☐	☐	☐	☐	☐	☐

3. Choose a number between "0, Not at all confident " and "10, Very confident" that describes how <u>confident</u> you feel about your knee when performing activities such as…

	0 Not at all confident	1	2	3	4	5	6	7	8	9	10 Very confident
…going up stairs?	☐	☐	☐	☐	☐	☐	☐	☐	☐	☐	☐
…kneeling on your knee?	☐	☐	☐	☐	☐	☐	☐	☐	☐	☐	☐
…going down stairs?	☐	☐	☐	☐	☐	☐	☐	☐	☐	☐	☐
…getting in/out of a car?	☐	☐	☐	☐	☐	☐	☐	☐	☐	☐	☐
…sitting down on a toilet?	☐	☐	☐	☐	☐	☐	☐	☐	☐	☐	☐
…walking on an uneven surface (such as a bumpy/broken sidewalk, sloping surface, etc.)?	☐	☐	☐	☐	☐	☐	☐	☐	☐	☐	☐
…getting up from a toilet?	☐	☐	☐	☐	☐	☐	☐	☐	☐	☐	☐

4. How often do you feel <u>confident</u> about your knee when performing your day-to-day activities?

Never	Rarely	Sometimes	Often	Always
☐	☐	☐	☐	☐

5. Choose a number between "0, Not at all stable" and "10, Very stable" that describes how physically <u>stable</u> your knee feels when doing activities such as…

	0 Not at all stable	1	2	3	4	5	6	7	8	9	10 Very stable
…going up stairs?	☐	☐	☐	☐	☐	☐	☐	☐	☐	☐	☐
… walking up a hill/ ramp/incline?	☐	☐	☐	☐	☐	☐	☐	☐	☐	☐	☐
… going down stairs?	☐	☐	☐	☐	☐	☐	☐	☐	☐	☐	☐
… going down a hill/ ramp/decline?	☐	☐	☐	☐	☐	☐	☐	☐	☐	☐	☐
… walking on slippery surfaces (such as wet grass, rainy streets, etc.)?	☐	☐	☐	☐	☐	☐	☐	☐	☐	☐	☐
… getting out of a car after driving or riding long distances?	☐	☐	☐	☐	☐	☐	☐	☐	☐	☐	☐

6. How often does your knee feel physically <u>stable</u> when performing your day-to-day activities?

Never	Rarely	Sometimes	Often	Always
☐	☐	☐	☐	☐

7. Choose a number between "0, Never" and "10, Always" that describes how often you modify or change the way you do activities such as…

	0 Never	1	2	3	4	5	6	7	8	9	10 Always
… bending down to the floor (to pick up an object, reach an item in a low cabinet, etc.)?	☐	☐	☐	☐	☐	☐	☐	☐	☐	☐	☐
… putting on your shoes?	☐	☐	☐	☐	☐	☐	☐	☐	☐	☐	☐
… going up stairs?	☐	☐	☐	☐	☐	☐	☐	☐	☐	☐	☐
… going down stairs?	☐	☐	☐	☐	☐	☐	☐	☐	☐	☐	☐
… getting in and out of a car?	☐	☐	☐	☐	☐	☐	☐	☐	☐	☐	☐

8. To what degree does your knee allow you to do the activities you want to do now?

Not at all	Slightly	Moderately	Very	Completely
☐	☐	☐	☐	☐

9. Overall, how satisfied are you with how your knee functions?

Very Dissatisfied	Dissatisfied	A little Dissatisfied	A little Satisfied	Satisfied	Very Satisfied
☐	☐	☐	☐	☐	☐
