# Supplementary material for: Translation and Linguistic Validation of the Patient's Knee Implant Performance (PKIP) into Japanese
Source: Adv Orthop. 2024 Apr 30;2024:6645361. doi: 10.1155/2024/6645361 (PMC11074845; doi:10.1155/2024/6645361)
Supplement: Supplementary Materials — Supplement 1: the original English version of the PKIP questionnaire for presurgical. Supplement 2: the original English version of the PKIP questionnaire for postsurgical. Supplement 3: the Japanese translation version of the PKIP questionnaire for presurgical. Supplement 4: the Japanese translation version of the PKIP questionnaire for postsurgical. [file 6645361.f1.zip › Supplement 2. English PKIP (post-surgery) (2).pdf]

PATIENT'S KNEE IMPLANT PERFORMANCE (PKIP) — Post Surgical

The questions below ask about ways in which your knee replacement is working with your body to improve your performance. Please select only one answer for each question.

---

1. How often are you aware of your knee?

Never	Rarely	Sometimes	Often	Always
☐	☐	☐	☐	☐

The following questions should be answered thinking of your knee replacement during the **last week**.

2. Overall, how satisfied are you with how your replacement knee feels when doing <u>everyday activities</u>?

Very Dissatisfied	Dissatisfied	A little Dissatisfied	A little Satisfied	Satisfied	Very Satisfied
☐	☐	☐	☐	☐	☐

3. Choose a number between "0, Not at all confident " and "10, Very confident" that describes how <u>confident</u> you feel about your knee when performing activities such as…

	0 Not at all confident	1	2	3	4	5	6	7	8	9	10 Very confident
…going up stairs?	☐	☐	☐	☐	☐	☐	☐	☐	☐	☐	☐
…kneeling on your knee?	☐	☐	☐	☐	☐	☐	☐	☐	☐	☐	☐
…going down stairs?	☐	☐	☐	☐	☐	☐	☐	☐	☐	☐	☐
…getting in/out of a car?	☐	☐	☐	☐	☐	☐	☐	☐	☐	☐	☐
…sitting down on a toilet?	☐	☐	☐	☐	☐	☐	☐	☐	☐	☐	☐
…walking on an uneven surface (such as a bumpy/broken sidewalk, sloping surface, etc.)?	☐	☐	☐	☐	☐	☐	☐	☐	☐	☐	☐
…getting up from a toilet?	☐	☐	☐	☐	☐	☐	☐	☐	☐	☐	☐

4. How often do you feel <u>confident</u> about your knee when performing your day-to-day activities?

Never	Rarely	Sometimes	Often	Always
☐	☐	☐	☐	☐

5. Choose a number between "0, Not at all stable" and "10, Very stable" that describes how physically <u>stable</u> your knee feels when doing activities such as…

	0 Not at all stable	1	2	3	4	5	6	7	8	9	10 Very stable
…going up stairs?	☐	☐	☐	☐	☐	☐	☐	☐	☐	☐	☐
… walking up a hill/ ramp/incline?	☐	☐	☐	☐	☐	☐	☐	☐	☐	☐	☐
… going down stairs?	☐	☐	☐	☐	☐	☐	☐	☐	☐	☐	☐
… going down a hill/ ramp/decline?	☐	☐	☐	☐	☐	☐	☐	☐	☐	☐	☐
… walking on slippery surfaces (such as wet grass, rainy streets, etc.)?	☐	☐	☐	☐	☐	☐	☐	☐	☐	☐	☐
… getting out of a car after driving or riding long distances?	☐	☐	☐	☐	☐	☐	☐	☐	☐	☐	☐

6. How often does your knee feel physically <u>stable</u> when performing your day-to-day activities?

Never	Rarely	Sometimes	Often	Always
☐	☐	☐	☐	☐

7. Choose a number between "0, Never" and "10, Always" that describes how often you modify or change the way you do activities such as…

	0 Never	1	2	3	4	5	6	7	8	9	10 Always
… bending down to the floor (to pick up an object, reach an item in a low cabinet, etc.)?	☐	☐	☐	☐	☐	☐	☐	☐	☐	☐	☐
… putting on your shoes?	☐	☐	☐	☐	☐	☐	☐	☐	☐	☐	☐
… going up stairs?	☐	☐	☐	☐	☐	☐	☐	☐	☐	☐	☐
… going down stairs?	☐	☐	☐	☐	☐	☐	☐	☐	☐	☐	☐
… getting in and out of a car?	☐	☐	☐	☐	☐	☐	☐	☐	☐	☐	☐

8. To what degree does your knee allow you to do the activities you want to do now?

Not at all	Slightly	Moderately	Very	Completely
☐	☐	☐	☐	☐

9. Overall, how satisfied are you with how your knee functions?

Very Dissatisfied	Dissatisfied	A little Dissatisfied	A little Satisfied	Satisfied	Very Satisfied
☐	☐	☐	☐	☐	☐
