# Supplementary material for: Translation and Linguistic Validation of the Patient's Knee Implant Performance (PKIP) into Japanese
Source: Adv Orthop. 2024 Apr 30;2024:6645361. doi: 10.1155/2024/6645361 (PMC11074845; doi:10.1155/2024/6645361)
Supplement: Supplementary Materials — Supplement 1: the original English version of the PKIP questionnaire for presurgical. Supplement 2: the original English version of the PKIP questionnaire for postsurgical. Supplement 3: the Japanese translation version of the PKIP questionnaire for presurgical. Supplement 4: the Japanese translation version of the PKIP questionnaire for postsurgical. [file 6645361.f1.zip › Supplement 3. Japanese PKIP (pre-surgery) (1).docx]

**Japanese PKIP questionnaire (pre-surgery)**


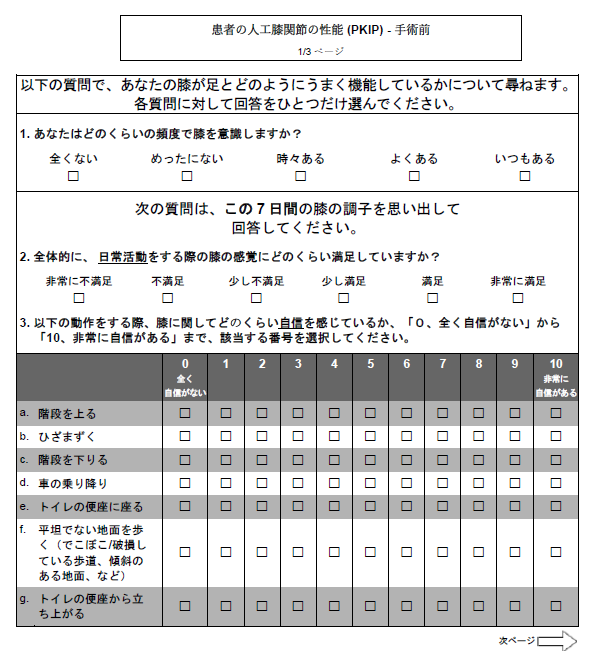


Note: Drawn on a smaller scale


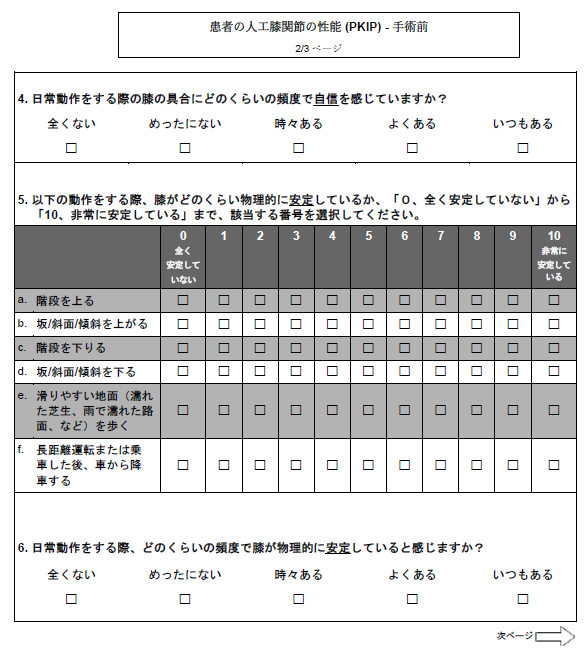


Note: Drawn on a smaller scale


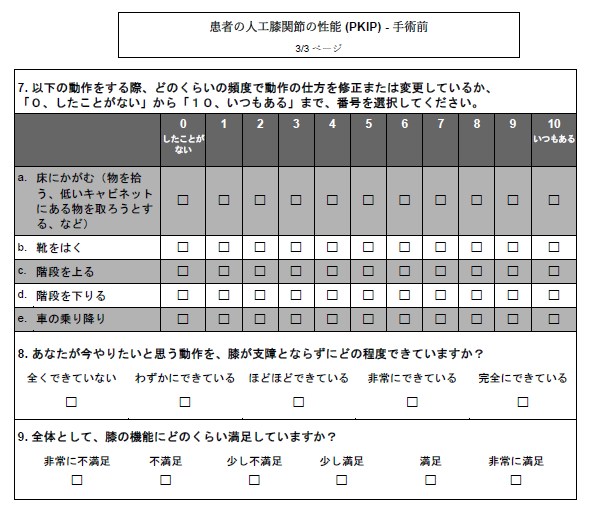


Note: Drawn on a smaller scale
